# Structural basis for recognition of 26RFa by the pyroglutamylated RFamide peptide receptor

**DOI:** 10.1038/s41421-024-00670-3

**Published:** 2024-06-04

**Authors:** Sanshan Jin, Shimeng Guo, Youwei Xu, Xin Li, Canrong Wu, Xinheng He, Benxun Pan, Wenwen Xin, Heng Zhang, Wen Hu, Yuling Yin, Tianwei Zhang, Kai Wu, Qingning Yuan, H. Eric Xu, Xin Xie, Yi Jiang

**Affiliations:** 1 Lingang Laboratory, Shanghai, China; 2 State Key Laboratory of Drug Research, Shanghai Institute of Materia Medica, Chinese Academy of Sciences, Shanghai, China; 3 University of Chinese Academy of Sciences, Beijing, China; 4 School of Pharmaceutical Science and Technology, Hangzhou Institute for Advanced Study, University of Chinese Academy of Sciences, Hangzhou, Zhejiang China; 5 The Shanghai Advanced Electron Microscope Center, Shanghai Institute of Materia Medica, Chinese Academy of Sciences, Shanghai, China; 6 School of Life Science and Technology, ShanghaiTech University, Shanghai, China; 7 Shandong Laboratory of Yantai Drug Discovery, Bohai Rim Advanced Research Institute for Drug Discovery, Yantai, Shandong China

**Keywords:** Cryoelectron microscopy, Molecular biology

## Abstract

The neuropeptide 26RFa, a member of the RF-amide peptide family, activates the pyroglutamylated RF-amide peptide receptor (QRFPR), a class A GPCR. The 26RFa/QRFPR system plays critical roles in energy homeostasis, making QRFPR an attractive drug target for treating obesity, diabetes, and eating disorders. However, the lack of structural information has hindered our understanding of the peptide recognition and regulatory mechanism of QRFPR, impeding drug design efforts. In this study, we determined the cryo-EM structure of the G_q_-coupled QRFPR bound to 26RFa. The structure reveals a unique assembly mode of the extracellular region of the receptor and the N-terminus of the peptide, and elucidates the recognition mechanism of the C-terminal heptapeptide of 26RFa by the transmembrane binding pocket of QRFPR. The study also clarifies the similarities and distinctions in the binding pattern of the RF-amide moiety in five RF-amide peptides and the RY-amide segment in neuropeptide Y. These findings deepen our understanding of the RF-amide peptide recognition, aiding in the rational design of drugs targeting QRFPR and other RF-amide peptide receptors.

## Introduction

RF-amide peptides are a family of neuropeptides widely present in most animal phyla^1,2^ which display a great sequence diversity while sharing a conserved C-terminal RF-amide sequence^1,3^. At present, five groups of RF-amide neuropeptides are identified in mammals. These neuropeptides include pyroglutamylated RF-amide peptide (QRFP), neuropeptide FF (NPFF), RF-amide-related peptide (RFRP or NPVF), prolactin-releasing peptide (PrRP), and kisspeptin groups. These neuropeptides act through five G protein-coupled receptors (GPCRs): pyroglutamine RF-amide peptide receptor (QRFPR, GPR103)^4^, neuropeptide FF receptor 1/2 (NPFF_1/2_R, GPR147/GPR74)^5^, prolactin-releasing peptide receptor (PrRPR, GPR10)^6^, and kisspeptin receptor (KISS1R, GPR54)^7^. These RF-amide peptides and their respective receptors play crucial roles in a wide range of neuroendocrine and behavioral functions, like modulation of feeding^8^, energy expenditure^9,10^, reproduction^11–14^, nociception^15^, and cardiovascular regulation^8^.

cDNA encoding the precursor or orthologue of 26RFa is detected in vertebrates, from fish^16^, and phasianidae^16^, to mammals^17^. In humans, 26RFa and its N-terminal extended mature peptide 43RFa are extracted from the human brain, and mRNA of QRFPR is detected in the hypothalamus, vestibular nuclei^4^, and other areas of the central nervous system. QRFPR is also detected in some peripheral organs^18,19^. Both 26RFa and 43RFa are endogenous ligands for QRFPR (GPR103)^4,18^. The amino acid sequences of 26RFa are conserved among mammalian species^20^, with 26RFa from rats being slightly more potent than that from humans in calcium mobilization^21^. Upon activation by 26RFa, QPFPR couples to G_q_ and G_i/o_ proteins^4,18^ and regulates a wide range of physiological functions, such as promoting sleep in zebrafish^22^, increasing food intake^8^ and insulin sensitivity^23^, modulating aldosterone secretion^24^, regulating bone formation^19^ and nociceptive transmission in rodent^25^. Selective agonists and antagonists of QRFPR may take effect in the treatment of metabolic imbalance (obesity, diabetes), eating disorders, and osteoporosis. The synthetic analog of the C-terminal heptapeptide of 26RFa targeting QRFPR showed long-lasting orexigenic effects in mice^21,26^, while the selective GPR103 antagonist demonstrated obvious anorexigenic activity in vivo^27^.

Considerable efforts have been dedicated to elucidating the recognition mechanism of 26RFa by QRFPR. Previous structure-activity analyses suggested that both the N- and C-termini of 26RFa participate in QRFPR recognition, with the C-terminus playing a particularly vital role in QRFPR activation^21^. Notably, the C-terminal heptapeptide was identified as the minimal active segment of 26RFa^21^. This segment served as a molecular scaffold for the development of low molecular weight peptides with enhanced potency and increased stability^21,28^. The small molecule Pyrrolo[2,3-c]pyridine, which mimics the C-terminal Arg-Phe motif of 26RFa, was developed as an antagonist of GPR103^27^. However, the absence of structural information on QRFPR has hampered our understanding of the recognition of 26RFa by QRFPR and impeded the rational design of drugs targeting QRFPR. Furthermore, the lack of any structural information on any family members of RF-amide peptide-bound receptors has posed challenges in understanding how the conserved RF-amide group of RF-amide neuropeptides regulates their specific receptors.

In this study, we determined the structure of the 26RFa–QRFPR–G_q_ complex utilizing the cryogenic electron microscopy (cryo-EM) technique. Combining structural and functional analyses, our findings unveil the unique assembly mode of the extracellular region of QRFPR and the N-terminus of 26RFa. The structure also provides insights into 26RFa recognition by QRFPR and suggests a general binding pattern of RF-amide peptides.

## Results

### The overall structure of the 26RFa–QRFPR–G_q_–ScFv16 complex

To facilitate the expression of the QRFPR–G_q_ complex, we introduced a cytochrome b562RIL (BRIL) at the N-terminus of the full-length wild-type (WT) human QRFPR^29,30^. A Gα_q_ chimera, designated as Gα_sqiN_, was engineered based on the mini-Gα_s_ scaffold with its N-terminus replaced by corresponding sequences of Gα_i1_ to facilitate the binding of scFv16. This Gα_sqiN_ chimera has been successfully used in the structure determination of GPCR–G_q_ complexes^31–33^. Hereinafter, G_q_ refers to Gα_sqiN_ chimera unless otherwise specified.

**Fig. 1:**
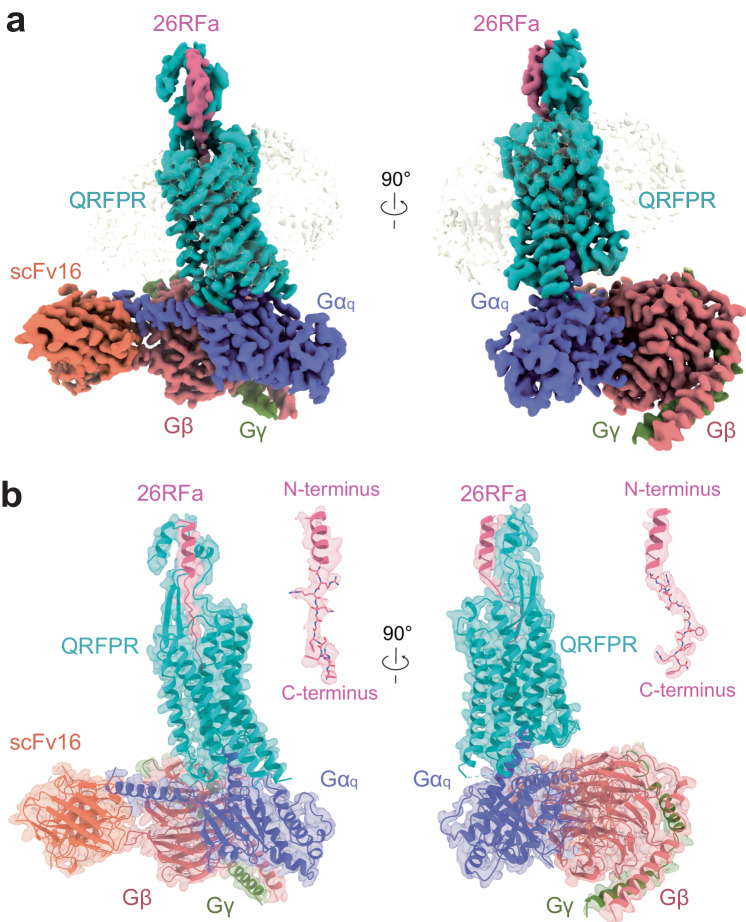
The cryo-EM structure of the 26RFa–QRFPR–G_q_–scFv16 complex. **a**, **b** The orthogonal views of the density map (**a**) and the model (**b**) of the 26RFa–QRFPR–G_q_–scFv16 complex are shown. The components of the complex are colored as indicated.

The NanoBiT tethering strategy was employed to stabilize the QRFPR–G_q_ complex^34^. Efficient assembly of the 26RFa–QRFPR–G_q_–scFv16 complex was achieved by incubating 26RFa with membranes from cells co-expressing the receptors, G_q_ heterotrimers, and scFv16 (Supplementary Fig. S1a). The structure of the complex was determined by cryo-EM at a global resolution of 2.73 Å (Fig. 1; Supplementary Fig. S1 and Table S1). The density map enabled model building for 26RFa and QRFPR containing residues E9–A346 (Fig. 1; Supplementary Fig. S2), except for two residues D249 and G258 in the intracellular loop 3 (ICL3). The side chains of the N-terminal region of 26RFa (Ser1–Lys19) and QRFPR (D9–L31) at the extracellular region of the complex were not defined due to ambiguous densities. QRFPR displays a canonical architecture of GPCR, consisting of seven transmembrane α-helices (TM1–TM7). 26RFa almost vertically inserts into the orthosteric binding pocket of QRFPR. The C-terminal tail of 26RFa is nestled within the TM core, and its N-terminus helix stretches outward and forms extensive interactions with the extracellular region of the receptor (Fig. 1).

### The unique assembly of the extracellular region of the 26RFa–QRFPR complex

Gly3–Tyr15 in 26RFa forms an α-helix, and the C-terminus displays as an extended loop. Our structure of 26RFa is slightly different from that of previous NMR analysis which showed that the α-helical region is formed between Gly6 and Tyr15^35^. The N-terminus and extracellular loop 2 (ECL2) of QRFPR, along with the N-terminal segment of 26RFa, assemble to constitute the extracellular region of the 26RFa–QRFPR complex.

**Fig. 2:**
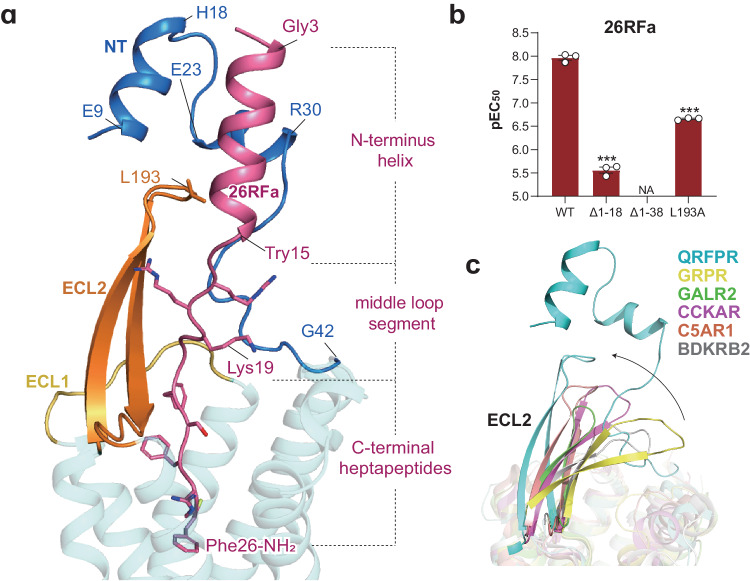
The unique assembly between the extracellular region of QRFPR and the N-terminus of 26RFa. **a** The architecture of the extracellular region of QRFRP bound to the N-terminus of 26RFa. NT, N-terminus. **b** Effects of mutations of the extracellular region of QRFPR on the potency of 26RFa-induced calcium mobilization. pEC_50_ values are shown as means ± SEM from three independent experiments performed in triplicate. ***P < 0.001. NA not activated. All data were analyzed by two-sided, one-way analysis of variance (ANOVA) with Tukey’s test. **c** Structure comparison of the extracellular region of QRFPR with those of other class A peptide-activated GPCRs. The orientation of ECL2 in QRFPR relative to other class A GPCRs is depicted by a black arrow. GPRP gastrin-releasing peptide receptor (PDB: 7W40), GALR2 galanin receptor 2 (PDB: 7WQ4), CCK_A_R cholecystokinin A receptor (PDB: 7EZH), C5AR1 complement component 5a receptor 1 (PDB: 7Y65), BDKRB2 bradykinin receptor B2 (PDB: 7F2O).

The N-terminus of QRFPR consists of two segments: a helical region (E9–R30) formed by two short perpendicular helices (E9–H18 and E23–R30) and a loop region (L31–G42) (Fig. 2a). These two short helices clip the N-terminus helix of 26RFa (Gly3–Tyr15) and cover the upper portion of ECL2. At the top of ECL2, L193 approaches the N-terminus of the receptor and the N-terminal helix of the peptide, potentially bridging ECL2 to both the receptor N-terminus and the peptide (Fig. 2a). The loop region of the receptor N-terminus and the β-hairpin of ECL2 are in line with the middle loop segment of peptide (Ser16–Arg19). These elements are oriented nearly vertically to the membrane and interact with ECL1 at the transmembrane interface (Fig. 2a).

Our functional analysis supports the crucial role of this unique extracellular assembly mode in 26RFa-induced QRFPR activation. Deleting the first short helix of the N-terminus (Δ1–18) resulted in a ~190-fold decrease of 26RFa potency (Fig. 2b; Supplementary Table S2). Truncation of the entire N-terminus (Δ1–38) abolished the peptide-induced QRFPR activity, highlighting the critical role of the QRFPR N-terminus (Fig. 2b). These findings align with a previous report that removing N-terminal 12 amino acids of 26RFa, which is the helical segment clipped by the loop region of the receptor N-terminus, led to a decrease in 26RFa activity by an order of magnitude^21^. Additionally, alanine substitution of L193 (L193A) at the N-terminus–ECL2–26RFa interface caused a remarkable decline in peptide activity (Fig. 2b). These structural observations and functional evidence highlight the importance of the assembly between the receptor’s extracellular region and the peptide in regulating the activity of QRFPR induced by 26RFa.

This assembly mode of the extracellular region in the 26RFa–QRFPR complex is unique compared to other reported class A GPCRs (Fig. 2c). In most class A GPCRs, the N-terminus is often short or lacks visible structural densities. In contrast, the main chains of the N-terminus of QRFPR (E9–G42) is defined in the EM density, probably due to its engagement with the N-terminus of 26RFa and ECL2 of the receptor (Fig. 2a). In addition, ECL2 in typical class A GPCRs tends to tilt towards the membrane and acts as a lid to cover the binding pocket, while the ECL2 in QRFPR is vertically oriented, opening a vestibule for the binding of 26RFa (Fig. 2c).

### Recognition of the C-terminal heptapeptide by QRFPR

**Fig. 3:**
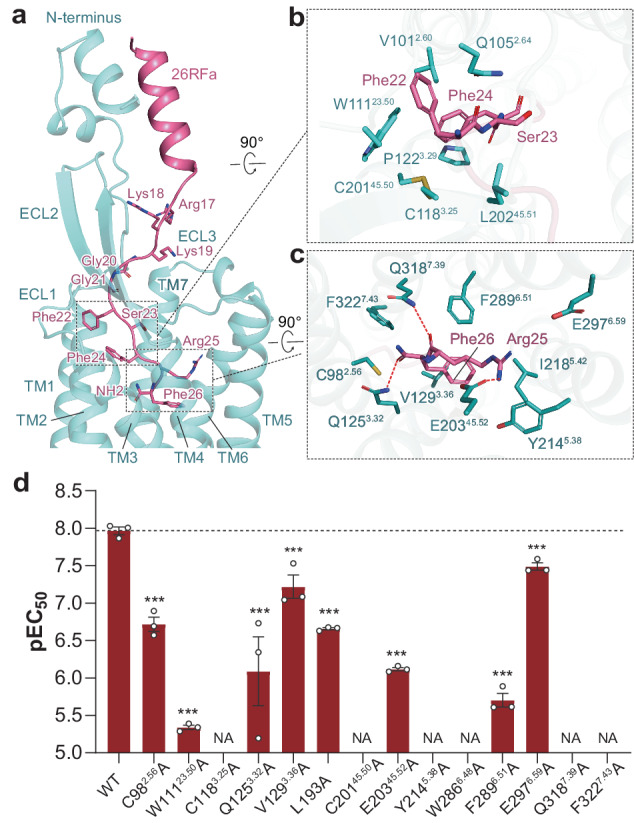
Recognition of the C-terminal heptapeptide of 26RFa by the transmembrane binding pocket of QRFPR. **a** The binding pose of 26RFa on QRFPR. The heptapeptide at the C-terminus of 26RFa occupies the transmembrane binding pocket of QRFPR. **b**, **c** Detail interactions between Phe22-Ser23-Phe24 (**b**) and Arg25-Phe26-amide segment (**c**) of 26RFa with residues of the transmembrane binding pocket in QRFPR. **d** Effects of mutations in the transmembrane binding pocket of QRFRP on the potency of 26RFa-mediated calcium mobilization. pEC_50_ values are shown as means ± SEM from three independent experiments performed in triplicate. ***P < 0.001. NA not activated. All data were analyzed by two-sided, one-way ANOVA with Tukey’s test.

Besides the N-terminal helix and middle loop segment of 26RFa interacting with the extracellular region of QRFPR, its C-terminal heptapeptide (Gly20–Phe26), the minimal active segment of 26RFa^21^, occupies the transmembrane binding pocket of QRFPR (Figs. 2a and 3a). Globally, the 26RFa segment (Gly20–Phe24) leans to TM2, but is far from TMs 5–7, leaving a large portion of unoccupied space lined by TMs 5–7 (Fig. 3a). Two glycines (Gly20, Gly21) are compactly packed into a crevice composed of residues on ECL1, ECL2, and the N-terminus (Fig. 3a). They moderately impact 26RFa activity, as substituting these two glycines of C-terminal heptapeptide with a bulkier alanine reduced the potency of 26RFa (20–26) by 4–5-fold^21^. The side chain of Phe22 forms an intramolecular hydrophobic interaction with Phe24, which leads to an extensive hydrophobic network with V101^2.60^, P122^3.29^, W111^23.50^, and the conserved disulfide bond between C118^3.25^ and C201^45.50^ (Fig. 3b). Alanine substitution of W111^23.50^ and two conserved cysteines notably hampered QRFPR activation (Fig. 3d; Supplementary Fig. S3a, b and Table S2), which coincides with the non-detectable activity of the C-terminal heptapeptide when substituting Phe24 with alanine or D-Phe^21^. These results demonstrated that Phe24 is crucial for 26RFa activity. In contrast, Ser23, whose side chain faces the spacious cavity of the peptide-binding pocket (Fig. 3a), shows a limited impact on 26RFa activity^28^.

The extreme C-terminus is deeply inserted into the TM helical core. The side chains of Arg25 and Phe26 point oppositely to those of Phe22 and Phe24 (Fig. 3a). The side chain of Arg25 builds a salt bridge with E203^45.52^, while its carbonyl group forms a hydrogen bond with Q318^7.39^. It also forms a cation–π interaction with Y214^5.38^ (Fig. 3c). The phenyl moiety of Phe26 is bordered by hydrophobic residues V129^3.36^, I218^5.42^, and F289^6.51^. The C-terminal amide group forms a hydrogen bond with Q125^3.22^. It is also coordinated by polar interaction with C98^2.56^ and within a distance for amide–π interaction with F322^7.43^ (Fig. 3c). All of these residues except I218^5.42^ are essential for 26RFa-induced receptor activation (Fig. 3d), which aligns with the observation that substituting Arg25 and Phe26 with alanine abolished the 26RFa activity^21^. The importance of the amide group is also supported by a previous report demonstrating that the non-amided form of 26RFa failed to activate QRFPR^18^. These findings reveal an essential role of the Arg25-Phe26-NH_2_ segment at the extreme C-terminus in 26RFa-induced QRFPR activation.

### Dynamics of RF26a–QRFPR interaction

To elucidate the dynamics of 26RFa–QRFPR interactions, we conducted four independent molecular dynamics (MD) simulations, each spanning 500 ns, for both apo and 26RFa-bound states of QRFPR. Our analysis revealed consistent hydrophobic interactions between 26RFa and specific residues (V101^2.60^, P122^3.29^, W111^23.50^, C118^3.25^, V129^3.36^, C201^45.50^, and F289^6.51^) throughout the simulations. Additionally, a stable salt bridge with E203^45.52^ and a hydrogen bond with Q318^7.39^ were observed, underscoring the importance of these interactions in 26RFa recognition by QRFPR (Supplementary Fig. S4a). These MD simulation results are consistent with our structural observation and functional analysis, providing strong support for our conclusions.

The examination of residue contacts during simulations, both in the absence and presence of 26RFa, highlighted unique contacts involving 26RFa–QRFPR interacting residues and exclusive to the 26RFa-bound trajectories. These interactions include C201^45.50^–Q184^4.64^, M180^4.60^–F123^3.30^, F97^2.56^–V101^2.60^–Q125^3.32^, and T102^2.61^–Q310^7.39^. Notably, 26RFa facilitates the bridging interactions between C201^45.50^ and Q184^4.64^. Furthermore, the presence of 26RFa within the TM bundle alters residue contacts, such as positioning Q125^3.32^ to interact with F97^2.56^ and V101^2.60^. These induced contacts, including M180^4.60^–F123^3.30^ and T102^2.61^–Q310^7.39^, are likely due to 26RFa’s influence on the spatial arrangement of the TM pocket. These findings suggest that 26RFa plays a significant role in receptor recognition and activation by modulating the interactions within the TM bundle (Supplementary Fig. S4b, c). These MD simulation results indicate the dynamics of 26RFa binding to QRFPR.

### Comparison of recognition modes of RF/RY-amide motifs by QPFR and neuropeptide Y receptors (NPYRs)

**Fig. 4:**
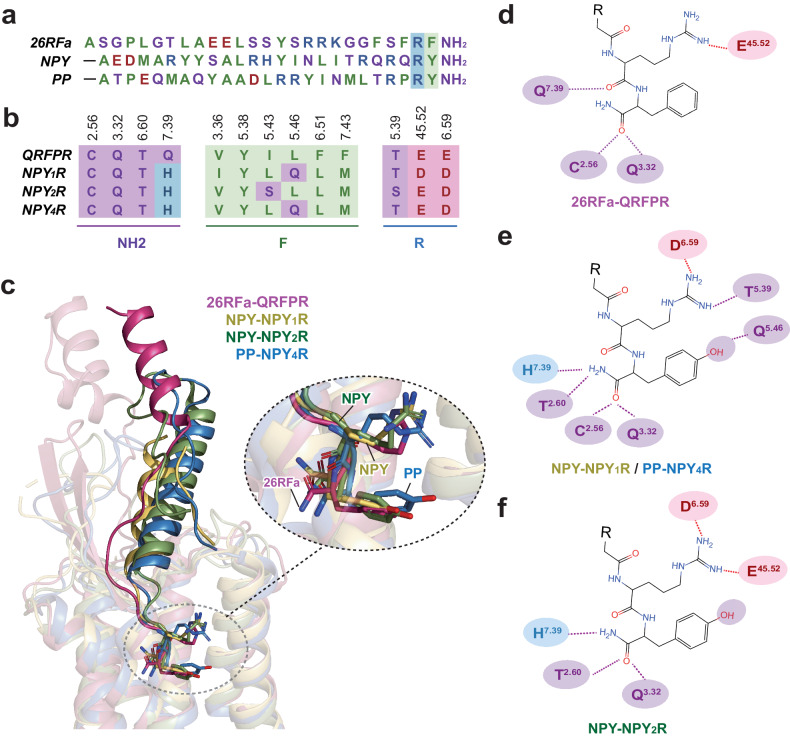
Comparison of recognition modes of peptide RF-amide segment by QRFPR and RY-amide segment by NPYRs. **a** Sequence alignment of 26RFa, NPY, and PP. The N-terminal sequence of NPY and PP are omitted. These peptides show similar RF-amide and RY-amide segments at their extreme C-terminus. **b** Sequence alignment of residues surrounding Arg25-Phe26-amide segment of 26RFa in QRFPR and cognate residues in NPY_1_R, NPY_2_R, and NPY_4_R. **c** Structural superposition of 26RFa in QRFPR, NPY in NPY_1_R/NPY_2_R (PDBs: 7X9A and 7X9B), and PP in NPY_4_R (PDB: 7X9C). **d**–**f** 2D representation of key interactions between the RF-amide segment in 26RFa (**d**), RY-amide in NPY (**e**) and PP (**f**), and their specific receptors. Hydrogen bonds and salt bridges are depicted as blue and red dashed lines, respectively. For residues presented in **a**, **b** and **d**–**f**, the light purple, blue, red, and green colors indicate polar, basic, acidic, and hydrophobic residues, respectively.

Neuropeptide Y (NPY) and pancreatic polypeptide (PP) have a similar amidated Arginine-Tyrosine (RY-amide) at its extreme C-terminus relative to the RF-amide motif in 26RFa (Fig. 4a). The binding pose of the N-terminus of 26RFa differs significantly from those of NPY in NPY_1_R (PDB: 7X9A), NPY_2_R (PDB: 7X9B), and PP in NPY_4_R (PDB: 7X9C). In contrast, the C-terminal RF-amide motif in 26RFa highly overlaps with RY-amide in NPY/PP (Fig. 4b). Sequence alignment also reveals highly conserved RF-amide/RY-amide binding sites for QRFPR and NPYRs (Fig. 4c).

Specifically, Arg25 and Arg35 in 26RFa and NPY/PP are exposed to a conserved polar environment that includes residues E^45.52^, T^5.39^, and D/E^6.59^ in QRFPR and NPYRs. However, subtle differences exist in the special binding interactions between these arginines and conserved polar residues (Fig. 4d–f). Side chains of Arg35 in NPY/PP form a conserved salt bridge with D^6.59^ and a hydrogen bond with T^5.39^ (NPY_1_R and NPY_4_R) or a salt bridge with E^45.52^ (NPY_2_R) (Fig. 4e, f). In 26RFa, Arg25 retains its salt bridge with E203^45.52^ but fails to build a salt bridge with E297^6.59^ (Fig. 3b). It is probably attributed to a greater distance between Arg25 and E297^6.59^ resulting from an outward movement of TM6 in QRFPR relative to those of NPYRs (Supplementary Fig. S5). In addition, the conserved polar residues surrounding the amide group of 26RFa and NPY/PP, such as C^2.57^, T^2.61^, Q^3.32^, and Q/H^7.39^, participate in forming intermolecular hydrogen bonds (Fig. 4d–f). These residues are critical for 26RFa and NPY/PP activities^36^, indicating similar receptor interaction modes of the amide group between 26RFa and NPY/PP.

Phe26 in 26RFa and Tyr36 in NPY/PP show distinct binding characteristics for QRFRP and NPYRs, respectively. The hydroxyl group of Tyr36 in NPY_1_R and NPY_4_R forms hydrogen bonds with Q^5.46^, an interaction crucial for NPY/PP activity^36^ (Fig. 4e). However, in NPY_2_R, this hydrogen bond is absent due to the substitution of glutamine with leucine at position 5.46, which lacks a hydrogen bond donor (Fig. 4c, f). Similarly, QRFPR also has a leucine at position 5.46, which cannot form a corresponding hydrogen bond with Phe26 in 26RFa. Furthermore, in contrast to the notable impact of Q^5.46^ on the NPY/PP activity for NPY_1_R and NPY_4_R, the presence of L^5.46^ in QRFPR and NPY_2_R shows negligible effects on the activities of the specific peptide^36^ (Supplementary Fig. S3). These findings suggest that Phe26 and Tyr36 in 26RFa and NPY/PP have distinct roles in activating their specific target receptors.

### Comparison of RF-amide segment recognition modes among RF-amide peptides

**Fig. 5:**
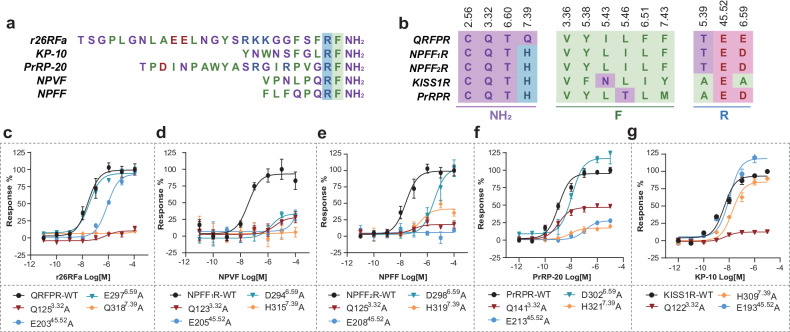
Comparison of recognition of RF-amide segment of RF-amide peptides by their specific receptors. **a** Sequence alignment of RF-amide peptides, which shares a conserved C-terminal RF-amide segment. **b** Sequence alignment of RF-amide-interacting residues across RF-amide peptide receptors. The light purple, blue, red, and green colors indicate polar, basic, acidic, and hydrophobic residues, respectively. **c**–**g** Effects of mutation in the RF-amide binding pocket on calcium mobilization. 26RFa/QRFPR (**c**); NPVF/NPFF_1_R (**d**); NPFF/NPFF_2_R (**e**); PrRP/PrRPR (**f**); KP-10/KISS1R (**g**).

The RF-amide C-terminal extremity is a molecular signature of 26RFa and other RF-amide peptides, such as NPFF, NPVF, PrRP, and kisspeptin (Fig. 5a). It was believed that the RF-amide segments within these peptides play a pivotal role in determining their bioactivity^37^. The RF-amide-interacting residues in QRFPR are relatively conserved across RF-amide peptide receptors (Fig. 5b). This raises a question of whether these RF-amide segments exhibit a common interaction pattern with their specific receptors. We thus introduced alanine substitutions on equivalent RF-amide-interacting residues in other RF-amide peptide receptors to evaluate their impacts on the activities of specific RF-amide peptides.

The highly conserved residues D/E^6.59^ and E^45.52^ across RF-amide peptide receptors are probably located surrounding arginine in the RF-amide peptides, as indicated by the structural model of the 26RFa–QRFPR complex (Fig. 3c). Our mutagenesis analyses showed that D/E^6.59^ is critical for the activities of NPVF and NPFF and also has a moderate impact on PrRP activity (Fig. 5d–f; Supplementary Fig. S3c and Tables S3–S5). These results are consistent with previous findings that D^6.59^ is important for the activation potency of NPFF1/2R^38^ and PrPR^39^. In contrast, mutating D^6.59^ to alanine almost did not affect 26RFa activity (Fig. 5c). Interestingly, in KISS1R, the positively charged D/E^6.59^ is replaced by an alanine (Fig. 5b; Supplementary Table S6), which is unable to form a salt bridge with the arginine in the RF-amide segment of KP-10, the smallest highly potent segment of kisspeptin^40,41^. As for E^45.52^, its substitution with alanine resulted in a remarkable decline of activity or potency of 26RFa, NPVF, NPFF, and PrRP towards QRFPR, NPFF_1_R, NPFF_2_R, and PrRPR, respectively (Fig. 5c–f). However, this mutation had a weak impact on KP-10 activity for KISS1R (Fig. 5g).

The phenylalanine in the RF-amide segment of 26RFa forms hydrophobic interactions with V129^3.36^ and F289^6.51^ in QRFPR, both of which substantially contribute to 26RFa activity (Fig. 3c, d). The valine at position 3.36 is identical, whereas the F289^6.51^ in QRFPR is substituted with leucine in other RF-amide peptide receptors (Fig. 5b). The mutation of F289^6.51^ to alanine resulted in a dramatic decrease in the activity of 26RFa (Fig. 3d), while a leucine substitution did not impact the 26RFa activity (Supplementary Fig. S3a). These findings highlight the importance of the hydrophobic interaction between Phe26 and F289^6.51^ for the activity of 26RFa and also suggest potential involvement of V^3.36^ and L^6.51^ in the activity of other RF-amide peptides towards their receptors.

In addition, Q123^3.32^ and Q318^7.39^ surrounding the amide group form hydrogen bonds with the RF-amide segment in 26RFa and are essential for the peptide activity (Fig. 3c, d). Sequence alignment shows that the glutamine at position 3.32 is conserved, whereas glutamine at position 7.39 in QRFPR is substituted with histidine in other RF-amide peptide receptors (Fig. 5b). Both Q^3.32^ and Q/H^7.39^ were critical for the activity of 26RFa, NPVF, NPFF, and PrRP (Fig. 5c–f). In the case of KP-10, Q^3.32^ also exhibited a remarkable impact to KP-10 activity. However, H^7.39^ showed only a minor effect (Fig. 5g). Collectively, in contrast to KP-10, the RF-amide segment is more crucial for the activity of other RF-amide peptides. This finding is supported by the fact that, in contrast to KP-10, the alanine substitution of arginine had a more significant impact on the activity of other RF-amide peptides^37,42^.

### Activation mechanism of QRFPR

Structural comparison of the 26RFa–QRFPR complex with its homologous receptor NPY_1_R in inactive (PDB: 5ZBQ) and active states (PDB: 7X9A) reveals a notable activation feature of QRFPR. Akin to the active NPY_1_R, the cytoplasmic end of TM6 of QRFPR undergoes a pronounced outward displacement in contrast to the inactive NPY_1_R, the hallmark of class A GPCR activation. Concomitantly, TM7 of QRFPR shows a slightly inward shift toward the core of the helical bundle (Supplementary Fig. S6a, b). These structural observations support the fact that the 26RFa-bound QRFPR is indeed in the active state.

Similar to the tyrosine in the RY-amide segment of NPY, the phenyl group of phenylalanine in the RF-amide moiety of 26RFa inserts into the helical core, enabling direct contact with W286^6.48^ (Supplementary Fig. S6c), the conserved toggle switch residue in class A GPCRs responsible for peptide-induced receptor activation^43,44^. The insertion may lead to a rotameric switch of W^6.48^ and induce the rearrangement of residues in other “micro-switch” motifs, including P^5.50^T^3.40^F^6.44^, N^7.49^P^7.50^xxY^7.53^, and E^3.49^R^3.50^Y^3.51^ (Supplementary Fig. S6d–f). These conformational changes are largely similar to those in the active NPY_1_R, indicating a shared activation mechanism between QRFPR and NPY_1_R.

### G_q_ coupling of QRFPR

The structure of the 26RFa–QRFPR–G_q_ complex provides a template for analyzing the G_q_ coupling mode of QRFPR. In comparison to G_q_-coupled ET_B_R (PDB: 8HCX) and CCK1R (PDB: 7MBY), QRFPR exhibits an overall conserved G_q_ coupling mode and shares two major G protein-coupling interfaces (Supplementary Fig. S7a, b). One primary interface is formed between the cytoplasmic cavity of receptor helices and the α5 helix of the Gα_q_ subunit. The extreme C-terminal residues in the α5 helix of the Gα_q_ subunit, known as “wavy hook” (Y358-N359-L360-V361), make extensive polar and hydrophobic interactions with the receptor (Supplementary Fig. S7c). The other hydrophobic interface is established between ICL2 of the receptor and a hydrophobic groove formed by the αN helix, α5 helix, and β2–β3 loop of the Gα_q_ subunit (Supplementary Fig. S7d). P150^34.50^ and F151^34.51^ in ICL2 anchor an extensive hydrophobic network with Gα_q_ residues (L34, V194, F343, and I350) (Supplementary Fig. S7d). Additionally, T158^ICL2^, W155^34.55^, and K154^34.54^ in ICL2 form hydrogen bonds with R31 and R32 in the Gα_q_ subunit (Supplementary Fig. S7d).

However, despite the overall similarity, QRFPR displays several distinct G_q_ coupling features compared to G_q_-coupled ET_B_R and CCK1R. It exhibits different relative positions of the α5 and αN helices, along with elongated cytoplasmic ends of TM5 and TM6 (Supplementary Fig. S7a, c). Residues on the extended helices of QRFPR, such as E240^5.64^, K245^5.69^, and K268^6.30^, form additional salt bridges with K347, D348, and Q352 in the α5 helix of the Gα_q_ subunit, respectively, further enhancing the stability of the complex (Supplementary Fig. S7c).

## Discussion

The 26RFa/QRFPR system plays critical roles in energy homeostasis. Uncovering the atomic details of 26RFa recognition and regulation of QRFPR is crucial for unraveling the function of QRFPR and developing effective treatments against obesity, diabetes, and eating disorders. Here, our structure of the 26RFa–QRFPR–G_q_ complex reveals a unique assembly mode of the extracellular region of QRFPR and the N-terminus of 26RFa, which differs from other class A GPCRs with reported structures. The unique organization mode is crucial for the recognition of 26RFa by QRFPR. The C-terminal heptapeptide of 26RFa inserts into the transmembrane binding pocket and is critical for QRFPR activation. The activation of QRFPR by 26RFa likely follows the “message-address” peptide binding model observed in other class A peptide-activated GPCRs^45^. In this model, the C-terminus of 26RFa, which determines peptide activity, serves as a “message”. The N-terminus of 26RFa retains the full potency to activate QRFPR, potentially by recognizing the extracellular region of the receptor and facilitating receptor activation, and is potentially responsible for peptide recognition and selectivity, representing an “address”. The specific interactions between the non-conserved “address” moiety of 26RFa and the non-conserved extracellular region of QRFPR compared to RF-amide peptides and their receptors may contribute to the high selectivity of 26RFa for QRFPR^46^. The model is also supported by previous findings that 26RFa (1–18), representing the N-terminal segment of native 26RFa lacking the C-terminal octapeptide, loses its ability to activate the receptor, while the C-terminal hepapeptide shows a remarkably decreased activity^21^.

In the helical bundle peptide-binding pocket, the C-terminal heptapeptide of 26RFa establishes an extensive interaction network with QRFPR. Unraveling the binding mode offers an opportunity for optimizing peptide activity. For example, Ser23 is exposed to a spacious area without significant interactions with neighboring residues in QRFPR. Substituting Ser23 with the bulkier norvaline led to a peptide analog that was 3-fold more potent than the native C-terminal heptapeptide of 26RFa^21^, which is supported by a slightly decreased free energy of [Nva^23^]-26RFa (–220.73 kcal/mol) compared with the native 26RFa (–220.29 kcal/mol), as determined using the MMGBSA approach.

The RF-amide segment at the extreme C-terminus of 26RFa is identical across other RF-amide peptides and similar to RY-amide group of NPY. The structural comparison reveals that the RF-amide moiety of 26RFa shares a similar binding mode with the RY-amide group of NPY for their specific receptors. Our sequence alignment and mutagenesis analyses also suggest largely conserved recognition modes of RF-amide peptides. Therefore, we propose a general binding pattern for RF-amide peptides in QRFPR, NPFF_1/2_R, and PrRPR, albeit with some distinctions in the case of KISS1R. In summary, our structural findings provide insights into the mechanisms of peptide recognition and activation of QRFPR and indicate a similar binding pattern for the RF-amide segment of RF-amide peptides across their specific receptors. These discoveries offer opportunities for the design of QRFPR-targeting drugs.

## Materials and methods

### Constructs

The gene encoding human WT QRFPR was codon-optimized for Hight Five (Hi5) insect cell expression. Full-length optimized QRFPR sequence was cloned into a modified pFastBac vector (Thermo Fisher) with a thermally stabilized N-terminal BRIL^29^ tag facilitating receptor expression and a C-terminal 8× His tag for receptor affinity purification. NanoBiT was split into a large subunit (LgBiT, 18 kDa) and a complementary small subunit (SmBiT, peptide, VTGYRLFEEIL). A homolog peptide of SmBiT, HiBiT (peptide 86, VSGWRLFKKIS), shows a high affinity for LgBiT^47^. We fused the LgBiT to the C-terminus of the receptor and the HiBiT with a 15-amino acid (15AA) polypeptide linker (GSSGGGGSGGGGSSG) at the C-terminus of Rat Gβ1. This NanoBiT tethering strategy^34^ was introduced to improve the structural homogeneity and stability of the QRFPR–G protein complex. The engineered Gα_q_ was designed based on the mini-Gα_s_ skeleton^48^. 18 amino acids from the N-terminus of Gα_q_ were replaced by the counterpart of Gα_i_ which was responsible for scFv16 binding^49^. Two dominant-negative mutations (corresponding to G203A and A326S)^50^ were incorporated to decrease the affinity of nucleotide binding to stabilize the Gαβγ complex. The engineered Gα_q_, Gβ1, bovine Gγ2, and scFv16 were cloned into the pFastBac vector, respectively.

### Expression and purification of complexes

Bac-to-Bac baculovirus was introduced in our expression system. Baculoviruses of FLAG-Bril-QRFPR (1–431)-LgBiT-H8, engineered Gα_q_, Gβ1-15AA-HiBiT, Gγ2, and scFv16 co-infected Hi5 insect cells (Invitrogen) in logarithmic phase at a 1:1:1:1:1 ratio. Hi5 cells were cultured in SIM-HF (SinoBiological) serum-free medium. Insect cells were scaled up and grown in suspension at 27 °C, rotating at 120 rpm, then harvested after 48 h and stored at –80 °C for use.

The frozen cells were thawed at 37 °C, resuspended in lysis buffer containing 20 mM HEPES, pH 7.5, 150 mM NaCl, 10% (v/v) glycerol, and EDTA-free protease inhibitor cocktail (TargetMol), and lysed by Dounce homogenizer. In the homogenization stage, 25 mU/mL Apyrase (Sigma-Aldrich) and 10 μM rat 26RFa (GenScript) were added into the cell lysate and incubated at room temperature for 1.5 h. The cell lysate was then solubilized in 0.5% (w/v) lauryl maltose neopentylglycol (LMNG, Anatrace), and 0.1% (w/v) cholesterol hemisuccinate (CHS, Anatrace) for 2 h at 4 °C. The supernatant was collected after centrifugation at 65,000× *g* for 35 min and incubated with TALON^®^ Metal Affinity Resin (TaKaRa) for 2 h at 4 °C. The resin was packed and washed with 30 column volumes of wash buffer (20 mM HEPES, pH 7.5, 150 mM NaCl, 0.01% (w/v) LMNG, and 0.002% CHS, 10 mM imidazole, 1 μM rat 26RFa) and finally eluted in the buffer containing 300 mM imidazole. The eluted sample was concentrated using an Amicon Ultra Centrifugal Filter (MWCO: 100 kDa) and purified by Superose 6 Increase 10/300 column (GE Healthcare) pre-equilibrated with buffer containing 20 mM HEPES, pH 7.5, 150 mM NaCl, 0.00075% (w/v) LMNG, 0.00025% (w/v) GDN (Anatrace) and 0.00015% CHS (1 μM 26RFa was used). Peak fractions were collected and concentrated for the cryo-EM study.

### Cryo-EM data collection

Cryo-EM grids were prepared with the Vitrobot Mark IV plunger (FEI) set to 6 °C and 100% humidity. Three microliters of the sample were applied to the glow-discharged gold R1.2/1.3 holey carbon grids. The sample was incubated for 10 s on the grids before blotting for 4.5 s (double-sided, blot force 1) and flash-frozen in liquid ethane immediately. For 26RF–QRFPR–G_q_ complex datasets, 3657 movies were collected on a Titan Krios equipped with a Falcon 4 direct electron detection device at 300 kV with a magnification of 165,000×, corresponding to a pixel size of 0.73 Å. Image acquisition was performed with EPU Software (FEI Eindhoven, the Netherlands). We collected a total of 36 frames accumulating to a total dose of 50 e^–^/Å^2^ over 2.5 s exposure.

### Cryo-EM image processing

MotionCor2 was used to perform the frame-based motion-correction algorithm to generate a drift-corrected micrograph for further processing^51,52^. All subsequent steps including contrast transfer function (CTF) estimation, particle picking and extraction, two-dimensional (2D) classification, ab-initio reconstruction, hetero refinement, non-uniform refinement, local refinement, and local resolution estimation were performed using cryoSPARC^53^.

For the dataset, 3657 dose-weighted micrographs were imported into cryoSPARC, and CTF parameters were estimated using patch-CTF. A blob picker was used for initial particle selection from a few micrographs followed by 2D classification to generate good templates. Subsequently, 2,397,272 particles were picked by template picker from the full set of micrographs and extracted using a pixel size of 2.92 Å. After two rounds of 2D classification, some of the classes that showed bad features were selected to generate three bad references by ab-initio reconstruction. emd_32313^54^ map was imported as a good reference. Using these references, the full set of particles was subjected to four rounds of heterogeneous refinement, resulting in a 3.32 Å map reconstructed from 99,325 particles. 20 classes of 2D templates were generated from the 3D maps. Subsequently, 2,449,719 particles were picked by template picker from the full set of micrographs and extracted using a pixel size of 2.92 Å. After one round of 2D classification, 1,850,060 particles were selected for further heterogenous refinement. After three rounds of heterogeneous refinement, 393,048 particles remained, and re-extracted using a pixel size of 0.73 Å. Following non-uniform refinement and local refinement, a map reached a resolution of 2.88 Å. Several rounds of heterogeneous refinement were conducted with updated reference maps, and 202,884 particles remained. Following non-uniform refinement and local refinement, the final map reached a resolution of 2.73 Å. We also performed postprocessing of the final maps with DeepEMhancer^55^

### Model building

A predicted QRFPR structure from AlphaFold2 was used as the starting reference model for receptor building^56^. Structures of Gα_q_, Gβ, Gγ, and the scFv16 were derived from PDB entry 8IUL^57^ and were rigid-body fit into the density. All models were fitted into the EM density map using UCSF Chimera^58^ followed by iterative rounds of manual adjustment and automated rebuilding in COOT^59^ and PHENIX^60^, respectively. The model was finalized by rebuilding in ISOLDE^61^ followed by refinement in PHENIX with torsion-angle restraints to the input model. The final model statistics were validated using Comprehensive validation (cryo-EM) in PHENIX^60^ and provided in Supplementary Table S1. All structural figures were prepared using Chimera^58^, Chimera X^62^, and PyMOL (Schrödinger, LLC.).

### Plasmid construction for functional assay

The gene encoding the WT QRFPR, PrPRR, KISS1R, NPFF_1_R, or NPFF_2_R was subcloned into the pcDNA3.0 vector with the addition of an N-terminal HA tag. All of the mutations used for functional studies were generated by QuickChange PCR and verified by DNA sequencing.

### Cell transfection

HEK293 cells were obtained from ATCC (Manassas, VA, USA) and cultured in DMEM supplemented with 10% (v/v) FBS, 100 mg/L penicillin, and 100 mg/L streptomycin in 5% CO_2_ at 37 °C. For transient transfection, ~2.5 × 10^6^ cells were mixed with 2 µg plasmids in 200 µL transfection buffer, and electroporation was carried out with a Scientz-2C electroporation apparatus (Scientz Biotech, Ningbo, China). The experiments were carried out 24 h after transfection.

### Calcium mobilization assay

For QRFPR, PrRPR, and KISS1R, HEK293 cells transfected with WT receptor or its mutants were seeded at a density of 4 × 10^4^ per well into 96-well culture plates and incubated for 24 h at 37 °C in 5% CO_2_. For NPFF_1_R and NPFF_2_R, plasmids were transfected into HEK293 cells stably expressing Gα_16_. On the next day, cells were incubated with 2 μmol/L Fluo-4 AM in HBSS supplemented with 5.6 mmol/L D-glucose and 250 μmol/L sulfinpyrazone at 37 °C for 45 min. After washing, cells were added with 50 μL HBSS and incubated at room temperature for 10 min, then 25 μL agonist buffer was dispensed into the well using a FlexStation III microplate reader (Molecular Devices), and intracellular calcium change was recorded at an excitation wavelength of 485 nm and an emission wavelength of 525 nm. EC_50_ and Emax values for each curve were calculated by Prism 8.0 software (GraphPad Software).

### Surface expression analysis

24 h after transfection, cells were washed with PBS, fixed with 4% PFA for 15 min, and then blocked with 2% BSA for 1 h. Next, cells were incubated with the polyclonal anti-HA (Sigma, H6908) overnight at 4 °C and then horseradish peroxidase (HRP)-conjugated anti-rabbit antibody (Cell Signaling Technology, 7074S) for 1 h at room temperature. Then cells were washed and incubated with 50 μL tetramethylbenzidine (Sigma, T0440) for 30 min, and the reaction was stopped with 25 μL TMB substrate stop solution (Beyotime, P0215). Absorbance at 450 nm was quantified using a FlexStation III microplate reader (Molecular Devices).

### MMGBSA binding free energy calculation

The 26RFa–QPRFR and [Nva^23^]-26RFa–QPRFR were constructed using the builder module in Maestro, Schrödinger Suite. Then the complex was prepared and minimized in Maestro protein preparation using OPLS4 force field. An implicit membrane of 44.5 Å height was placed according to the coordinates of helices. Then, MMGBSA with OPLS4 force field was used to evaluate the binding free energy of 26RFa and [Nva^23^]-26RFa towards QPRFR.

### MD simulation

The apo system was generated by removing 26RFa and G proteins prior to simulation, while holo system only has its G proteins removed. Protonation states of amino acid residues were determined using Propka3 software^63^. The CHARMM-GUI platform facilitated the integration of these structures into a 75 Å × 75 Å POPC lipid bilayer, which was then encapsulated by a 15 Å aqueous layer. The simulation environments were adjusted to a 0.15 mol/L NaCl concentration, with the addition of counterions to maintain electrochemical neutrality.

MD simulations employed the FF19SB force field for modeling amino acid interactions and the lipid21 force field for lipids^64,65^. A seven-step equilibration process recommended by CHARMM-GUI was followed, involving gradual minimization and relaxation of constraints to stabilize the systems.

Four independent production runs, each extending to 500 ns, were conducted for each system using the pmemd.cuda module in Amber20, under the NPT ensemble at 303.15 K and 1 atm^66^. Long-range electrostatic interactions were managed using the Particle Mesh Ewald method, and short-range electrostatic and van der Waals interactions were treated with a 12 Å cutoff, featuring a smooth transition between 10 Å and 12 Å. The interaction analysis was conducted by ProLIF^67^. The cluster and contact analysis was accomplished by CPPTRAJ^68^.

### Supplementary information

Supplementary information

## Data Availability

The cryo-EM density map and the atomic model coordinates for the 26RFa–QRFPR–G_q_–scFv16 complex have been deposited in the Electron Microscopy Data Bank with accession code EMD-37944 and the Protein Data Bank with accession code 8WZ2.

## References

[1] Walker RJ, Papaioannou S, Holden-Dye L (2009). A review of FMRFamide- and RFamide-like peptides in metazoa. Invert. Neurosci..

[2] Jékely G (2013). Global view of the evolution and diversity of metazoan neuropeptide signaling. Proc. Natl. Acad. Sci. USA.

[3] Espinoza E, Carrigan M, Thomas SG, Shaw G, Edison AS (2000). A statistical view of FMRFamide neuropeptide diversity. Mol. Neurobiol..

[4] Jiang Y (2003). Identification and characterization of a novel RF-amide peptide ligand for orphan G-protein-coupled receptor SP9155. J. Biol. Chem..

[5] Allard M, Geoffre S, Legendre P, Vincent JD, Simonnet G (1989). Characterization of rat spinal cord receptors to FLFQPQRFamide, a mammalian morphine modulating peptide: a binding study. Brain Res..

[6] Langmead CJ (2000). Characterization of the binding of [(125)I]-human prolactin releasing peptide (PrRP) to GPR10, a novel G protein coupled receptor. Br. J. Pharm..

[7] de Roux N (2003). Hypogonadotropic hypogonadism due to loss of function of the KiSS1-derived peptide receptor GPR54. Proc. Natl. Acad. Sci. USA.

[8] Takayasu S (2006). A neuropeptide ligand of the G protein-coupled receptor GPR103 regulates feeding, behavioral arousal, and blood pressure in mice. Proc. Natl. Acad. Sci. USA.

[9] Cázarez-Márquez F (2021). Role of central kisspeptin and RFRP-3 in energy metabolism in the male Wistar rat. J. Neuroendocrinol..

[10] Chartrel N (2016). The neuropeptide 26RFa (QRFP) and its role in the regulation of energy homeostasis: a mini-review. Front. Neurosci..

[11] Clarke IJ, Qi Y, Puspita Sari I, Smith JT (2009). Evidence that RF-amide related peptides are inhibitors of reproduction in mammals. Front. Neuroendocrinol..

[12] León S (2014). Physiological roles of gonadotropin-inhibitory hormone signaling in the control of mammalian reproductive axis: studies in the NPFF1 receptor null mouse. Endocrinology.

[13] Mamgain A (2021). RFamide-related peptide neurons modulate reproductive function and stress responses. J. Neurosci..

[14] Sari IP, Rao A, Smith JT, Tilbrook AJ, Clarke IJ (2009). Effect of RF-amide-related peptide-3 on luteinizing hormone and follicle-stimulating hormone synthesis and secretion in ovine pituitary gonadotropes. Endocrinology.

[15] Ayachi S, Simonin F (2014). Involvement of mammalian RF-amide peptides and their receptors in the modulation of nociception in rodents. Front. Endocrinol..

[16] Ukena K (2010). Identification, localization, and function of a novel avian hypothalamic neuropeptide, 26RFa, and its cognate receptor, G protein-coupled receptor-103. Endocrinology.

[17] Chartrel N (2003). Identification of 26RFa, a hypothalamic neuropeptide of the RFamide peptide family with orexigenic activity. Proc. Natl. Acad. Sci. USA.

[18] Fukusumi S (2003). A new peptidic ligand and its receptor regulating adrenal function in rats. J. Biol. Chem..

[19] Baribault H (2006). The G-protein-coupled receptor GPR103 regulates bone formation. Mol. Cell. Biol..

[20] Ukena K, Vaudry H, Leprince J, Tsutsui K (2011). Molecular evolution and functional characterization of the orexigenic peptide 26RFa and its receptor in vertebrates. Cell Tissue Res..

[21] Le Marec O (2011). Structure-activity relationships of a series of analogues of the RFamide-related peptide 26RFa. J. Med. Chem..

[22] Chen A (2016). QRFP and its receptors regulate locomotor activity and sleep in zebrafish. J. Neurosci..

[23] El Mehdi M (2022). The 26RFa (QRFP)/GPR103 neuropeptidergic system in mice relays insulin signalling into the brain to regulate glucose homeostasis. Diabetologia.

[24] Ramanjaneya M (2013). QRFP induces aldosterone production via PKC and T-type calcium channel-mediated pathways in human adrenocortical cells: evidence for a novel role of GPR103. Am. J. Physiol. Endocrinol. Metab..

[25] Yamamoto T, Wada T, Miyazaki R (2008). Analgesic effects of intrathecally administered 26RFa, an intrinsic agonist for GPR103, on formalin test and carrageenan test in rats. Neuroscience.

[26] Neveu C (2012). Rational design of a low molecular weight, stable, potent, and long-lasting GPR103 aza-beta3-pseudopeptide agonist. J. Med. Chem..

[27] Georgsson J (2014). GPR103 antagonists demonstrating anorexigenic activity in vivo: design and development of pyrrolo[2,3-c]pyridines that mimic the C-terminal Arg-Phe motif of QRFP26. J. Med. Chem..

[28] Neveu C (2012). Rational design of a low molecular weight, stable, potent, and long-lasting GPR103 aza-β3-pseudopeptide agonist. J. Med. Chem..

[29] Chun E (2012). Fusion partner toolchest for the stabilization and crystallization of G protein-coupled receptors. Structure.

[30] Zhuang Y (2021). Structural insights into the human D1 and D2 dopamine receptor signaling complexes. Cell.

[31] Kim K (2020). Structure of a hallucinogen-activated Gq-coupled 5-HT(2A) serotonin receptor. Cell.

[32] Wang Y (2021). Molecular recognition of an acyl-peptide hormone and activation of ghrelin receptor. Nat. Commun..

[33] Yin YL (2021). Molecular basis for kinin selectivity and activation of the human bradykinin receptors. Nat. Struct. Mol. Biol..

[34] Duan J (2020). Cryo-EM structure of an activated VIP1 receptor-G protein complex revealed by a NanoBiT tethering strategy. Nat. Commun..

[35] Thuau R (2005). Structural studies on 26RFa, a novel human RFamide-related peptide with orexigenic activity. Peptides.

[36] Tang T (2022). Receptor-specific recognition of NPY peptides revealed by structures of NPY receptors. Sci. Adv..

[37] Findeisen, M., Rathmann, D. Beck-Sickinger, A. G. RFamide peptides: structure, function, mechanisms and pharmaceutical potential. *Pharmaceuticals* **4**, 1248–1280 (2011).

[38] Findeisen M, Rathmann D, Beck-Sickinger AG (2011). Structure-activity studies of RFamide peptides reveal subtype-selective activation of neuropeptide FF1 and FF2 receptors. ChemMedChem.

[39] Rathmann D (2012). Ligand-mimicking receptor variant discloses binding and activation mode of prolactin-releasing peptide. J. Biol. Chem..

[40] Ohtaki T (2001). Metastasis suppressor gene KiSS-1 encodes peptide ligand of a G-protein-coupled receptor. Nature.

[41] Gutiérrez-Pascual E (2009). In vivo and in vitro structure-activity relationships and structural conformation of Kisspeptin-10-related peptides. Mol. Pharm..

[42] Orsini MJ (2007). Metastin (KiSS-1) mimetics identified from peptide structure-activity relationship-derived pharmacophores and directed small molecule database screening. J. Med. Chem..

[43] García-Nafría J, Nehmé R, Edwards PC, Tate CG (2018). Cryo-EM structure of the serotonin 5-HT(1B) receptor coupled to heterotrimeric G(o). Nature.

[44] Maeda S, Qu Q, Robertson MJ, Skiniotis G, Kobilka BK (2019). Structures of the M1 and M2 muscarinic acetylcholine receptor/G-protein complexes. Science.

[45] Tikhonova IG, Gigoux V, Fourmy D (2019). Understanding peptide binding in class A G protein-coupled receptors. Mol. Pharm..

[46] Elhabazi K (2013). Endogenous mammalian RF-amide peptides, including PrRP, kisspeptin and 26RFa, modulate nociception and morphine analgesia via NPFF receptors. Neuropharmacology.

[47] Dixon AS (2016). NanoLuc complementation reporter optimized for accurate measurement of protein interactions in cells. ACS Chem. Biol..

[48] Nehmé R (2017). Mini-G proteins: Novel tools for studying GPCRs in their active conformation. PLoS One.

[49] Maeda S (2020). Structure and selectivity engineering of the M(1) muscarinic receptor toxin complex. Science.

[50] Liu P (2016). The structural basis of the dominant negative phenotype of the Gαi1β1γ2 G203A/A326S heterotrimer. Acta Pharm. Sin..

[51] Zheng SQ (2017). MotionCor2: anisotropic correction of beam-induced motion for improved cryo-electron microscopy. Nat. Methods.

[52] Rohou A, Grigorieff N (2015). CTFFIND4: Fast and accurate defocus estimation from electron micrographs. J. Struct. Biol..

[53] Punjani A, Rubinstein JL, Fleet DJ, Brubaker MA (2017). cryoSPARC: algorithms for rapid unsupervised cryo-EM structure determination. Nat. Methods.

[54] You C (2022). Structural insights into the peptide selectivity and activation of human neuromedin U receptors. Nat. Commun..

[55] Sanchez-Garcia R (2021). DeepEMhancer: a deep learning solution for cryo-EM volume post-processing. Commun. Biol..

[56] Tunyasuvunakool K (2021). Highly accurate protein structure prediction for the human proteome. Nature.

[57] Wu C (2023). Ligand-induced activation and G protein coupling of prostaglandin F(2α) receptor. Nat. Commun..

[58] Pettersen EF (2004). UCSF Chimera-a visualization system for exploratory research and analysis. J. Comput. Chem..

[59] Emsley P, Cowtan K (2004). Coot: model-building tools for molecular graphics. Acta Crystallogr. D Biol. Crystallogr..

[60] Adams PD (2004). Recent developments in the PHENIX software for automated crystallographic structure determination. J. Synchrotron Radiat..

[61] Croll TI (2018). ISOLDE: a physically realistic environment for model building into low-resolution electron-density maps. Acta Crystallogr. D Struct. Biol..

[62] Pettersen EF (2021). UCSF ChimeraX: Structure visualization for researchers, educators, and developers. Protein Sci..

[63] Olsson MHM, Søndergaard CR, Rostkowski M, Jensen JH (2011). PROPKA3: consistent treatment of internal and surface residues in empirical pKa predictions. J. Chem. Theory Comput..

[64] Tian C (2020). ff19SB: amino-acid-specific protein backbone parameters trained against quantum mechanics energy surfaces in solution. J. Chem. Theory Comput..

[65] Dickson CJ, Walker RC, Gould IR (2022). Lipid21: complex lipid membrane simulations with AMBER. J. Chem. Theory Comput..

[66] Salomon-Ferrer R, Götz AW, Poole D, Le Grand S, Walker RC (2013). Routine microsecond molecular dynamics simulations with AMBER on GPUs. 2. explicit solvent particle mesh ewald. J. Chem. Theory Comput..

[67] Bouysset C, Fiorucci S (2021). ProLIF: a library to encode molecular interactions as fingerprints. J. Cheminform.

[68] Roe DR, Cheatham TE (2013). PTRAJ and CPPTRAJ: software for processing and analysis of molecular dynamics trajectory data. J. Chem. Theory Comput..

