# Supplementary material for: Structural basis for recognition of 26RFa by the pyroglutamylated RFamide peptide receptor
Source: Cell Discov. 2024 Jun 4;10:58. doi: 10.1038/s41421-024-00670-3 (PMC11148045; doi:10.1038/s41421-024-00670-3)
Supplement: Supplementary file 1 — Supplementary information [file 41421_2024_670_MOESM1_ESM.pdf]

Supplementary Information

Structure basis for recognition of 26RFa by the pyroglutamylated RFamide peptide receptor

Sanshan Jin[#], Shimeng Guo[#], Youwei Xu[#], Xin Li[#], Canrong Wu[#], Xinheng He, Benxun Pan, Wenwen Xin, Heng Zhang, Wen Hu, Yuling Yin, Tianwei Zhang, Kai Wu, Qingning Yuan, H. Eric Xu*, Xin Xie*, Yi Jiang*

This file includes:

Supplementary Figures S1-7

Figure Legends

Supplementary Tables S1-6

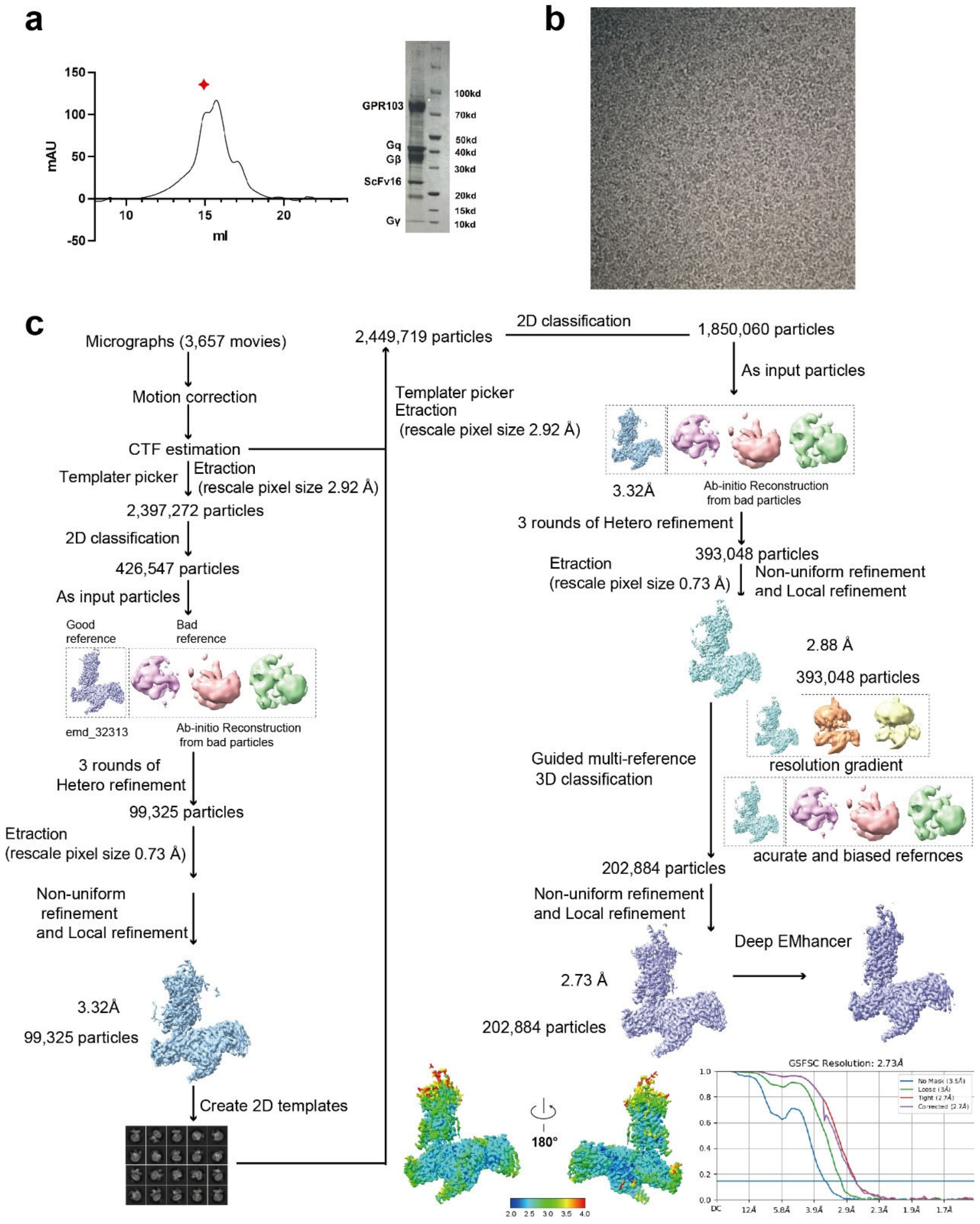

Supplementary Fig. S1 Purification and cryo-EM data processing of the 26RF-QRFPR-G$_q$-scFv16 complex. a Representative size-exclusion chromatography elution profile and SDS-PAGE analysis of the 26RF-QRFPR-G$_q$-scFv16 complex. The elution peak of the complex monomer is indicated by a red star. **b** Representative micrographs of the 26RF-QRFPR-G$_q$-scFv16 complex from CTF estimation. **c** The flowchart of data processing for the 26RF-QRFPR-G$_q$-scFv16 complex. Detailed descriptions can be found in the Methods section.

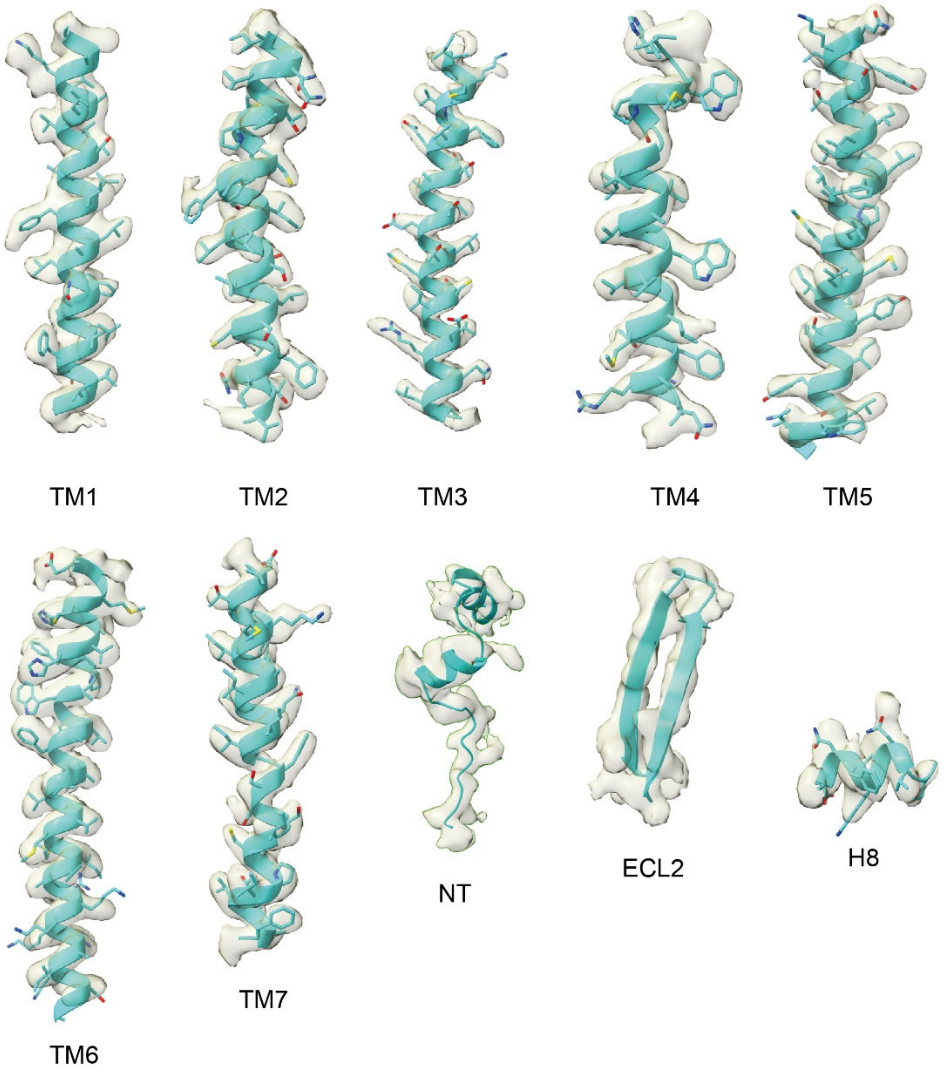

Supplementary Fig. S2 Representative densities of QRFPR in the 26RF-QRFPR-G$_q$-scFv16 complex. Densities of seven transmembrane helices, helix 8, N-terminus, and ECL2 are shown.

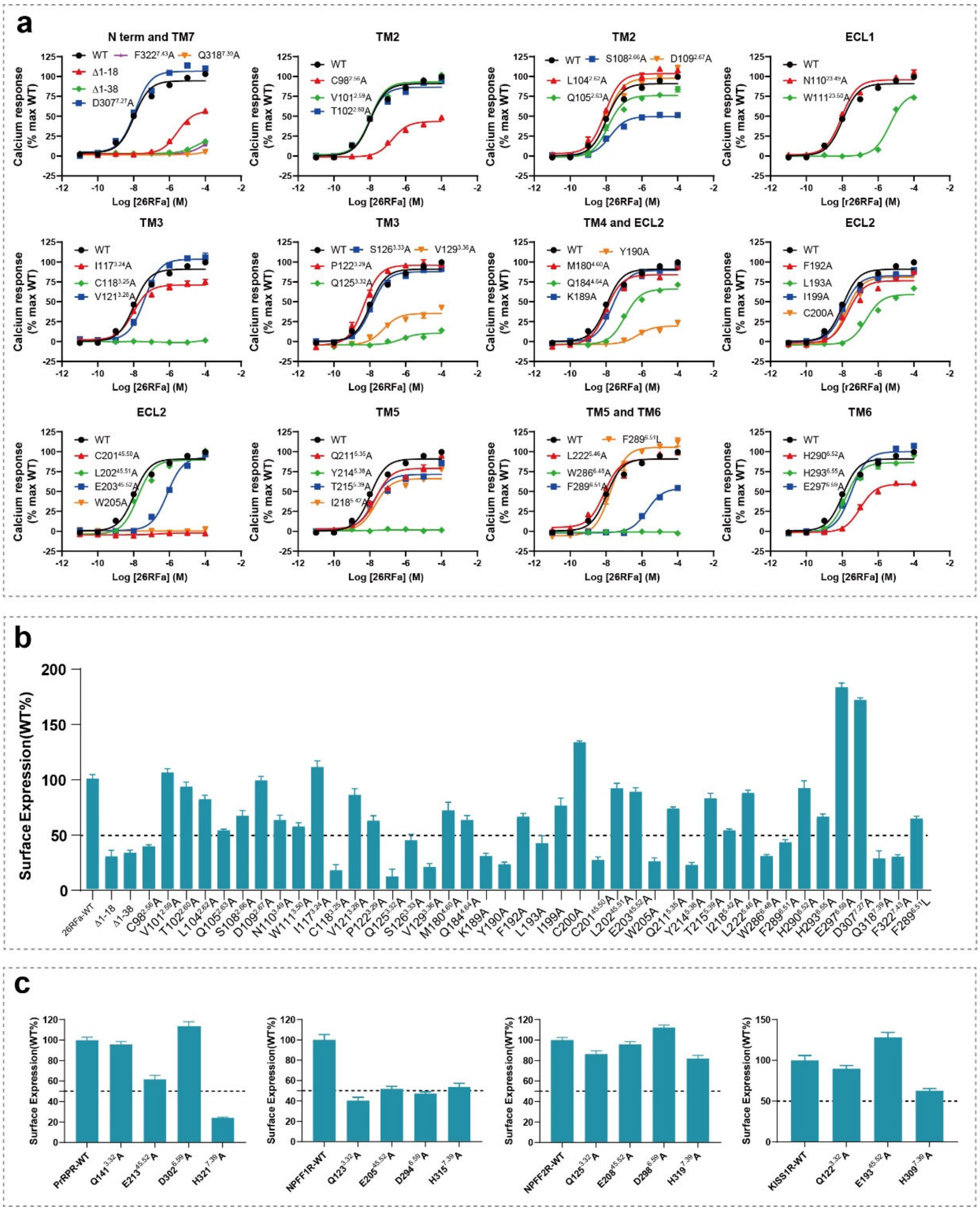

Supplementary Fig. S3 Effects of residue mutations in the QRFPR on 26RFa activity. a Dose-response curves of 26RFa on mutants of QRFPR. **b** Surface expression of QRFPR mutants. **c** Surface expression of critical residues in the RF-amide binding residues.

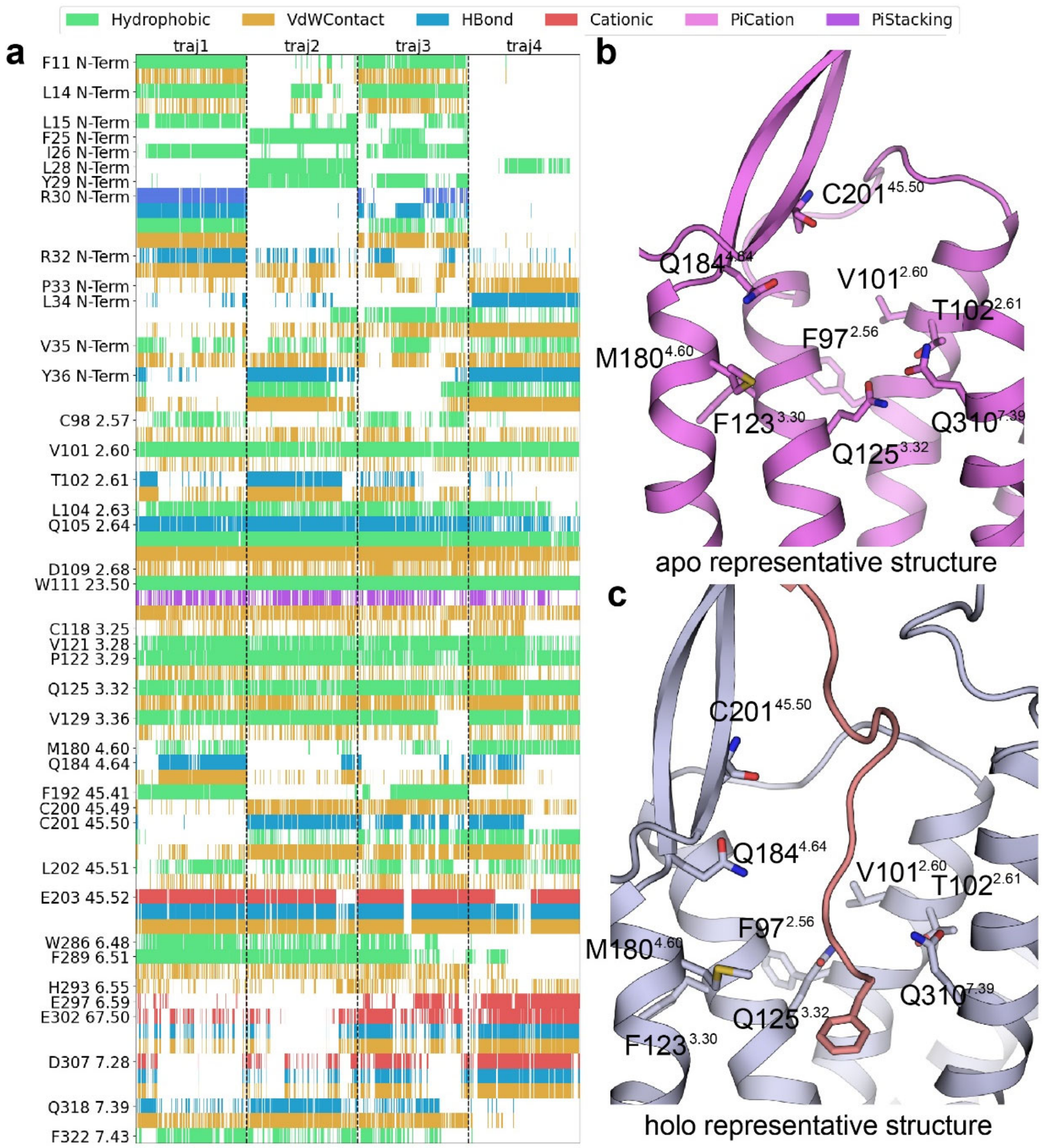

Supplementary Fig. S4 The dynamics of 26RFa and QRFPR interface. a The barcode plot mapping the interactions between 26RFa and QRFPR across four independent MD simulations over 500 ns. The types of interactions between 26RFa and QRFPR are detailed on the top. Only the interactions existing in more than 30% snapshots were shown. **b,c** representative structures from both apo (**b**) and 26RFa-QRFPR (**c**) trajectories. The residues that partake in various interactions upon 26RFa binding are depicted as sticks in the representative structures.

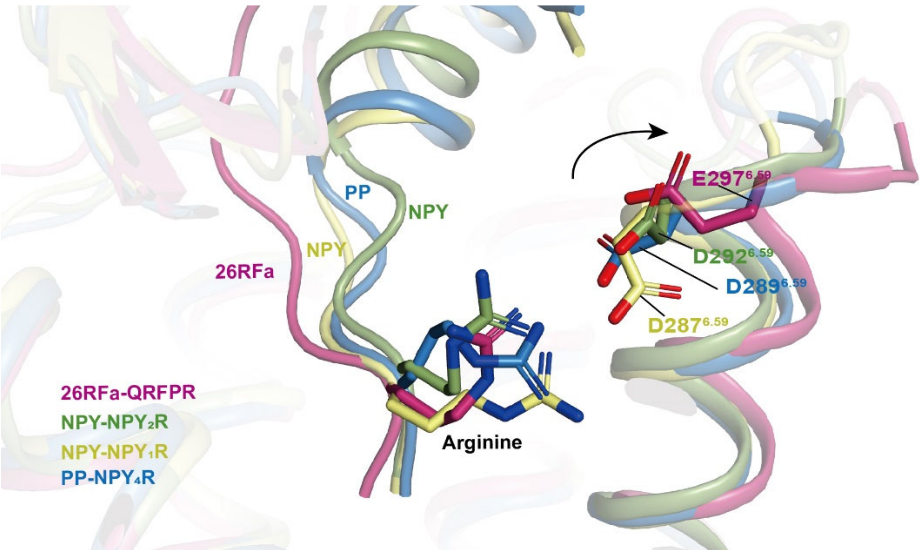

Supplementary Fig. S5 Comparison of interactions between the arginine in the RF/RY-amide motifs in 26RFa/NPY/PP and D/E$^{6.59}$. The TM6 in QRFPR shows an outward movement relative to NPY bound NPY_1R (PDB: 7X9A) and NPY_2R (PDB: 7X9B), and PP bound NPY_4R (PDB: 7X9C). Thus, in contrast to $D^{6.59}$ in NPYRs, the residue $E^{6.59}$ in QRFPR is positioned far from forming a salt bridge with the conserved arginine in the RF-amide motif.

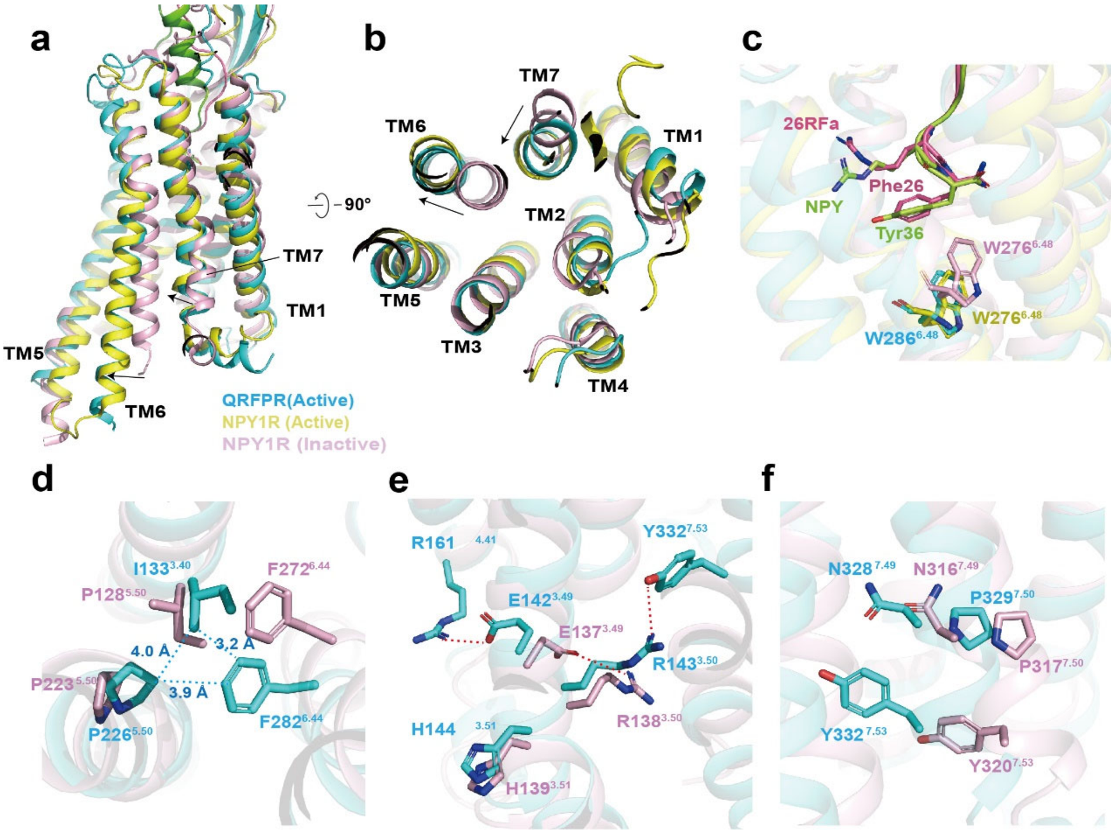

Supplementary Fig. S6 Activation mechanism of QRFPR by 26RFa. a, b Structural comparison of the 26RFa bound QRFPR in the active state with inactive and active neuropeptide Y receptor 1 (NPY$_1$R, PDB: 5ZBQ and 7X9A) in the side view (**a**) and bottom view (**b**). The movement orientations of the cytoplasmic ends of TM6 and TM7 in QRFPR relative to inactive NPY$_1$R are indicated by black arrows. **c** Arg26 and Tyr 36 at the C-terminus of 26RFa and NPY induces the movement of toggle switch residue W$^{6.48}$ in QRFPR and NPY$_1$R. The antagonist in the inactive NPY$_1$R structure is omitted for a clear representation. **d-f** Conformational comparisons of conserved micro-switches between QRFPR and the inactive NPY$_1$R. The conformation arrangements of residues in the PIF (**d**), DRY (**e**), and NPxxY (**f**) motifs are shown.

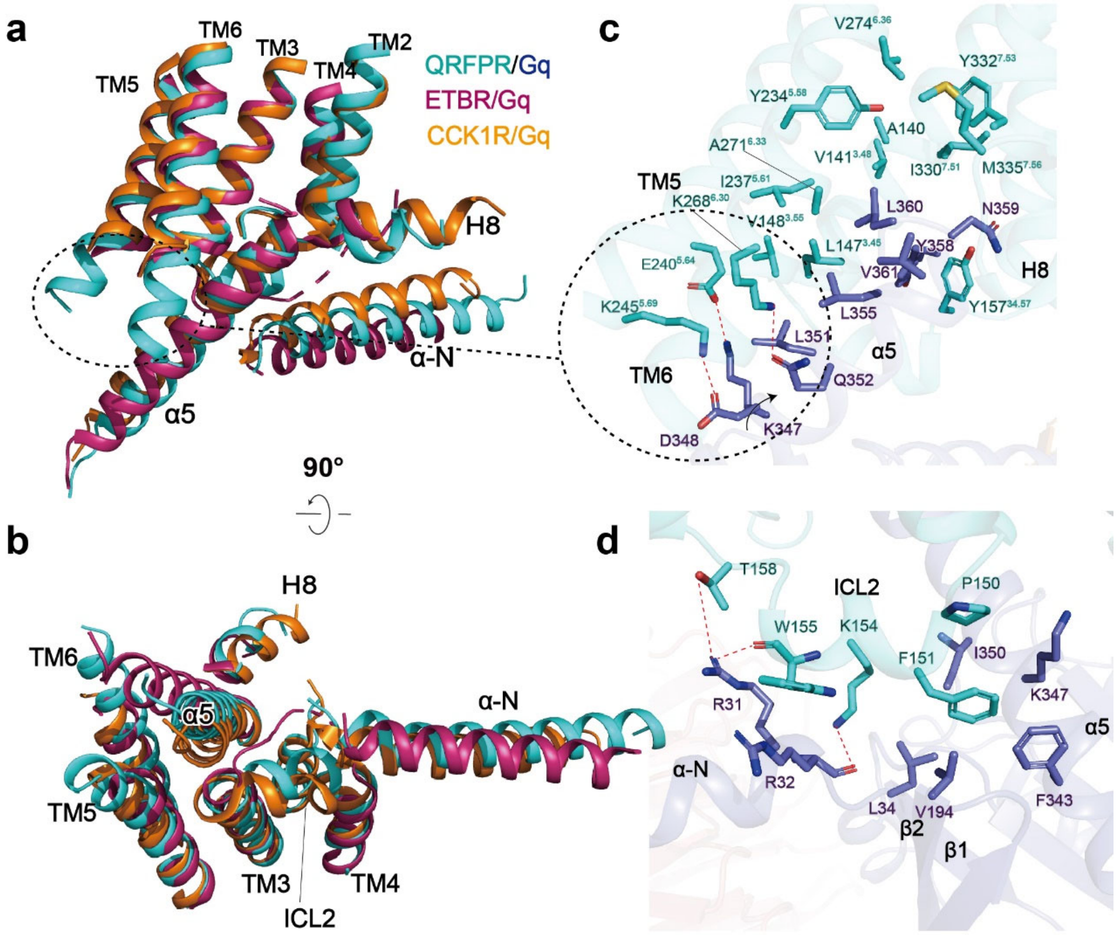

Supplementary Fig. S7 G_q coupling of QRFPR. a, b Structure alignment of G_q bound QRFPR, ET_BR (PDB:8HCX) and CCK1R (PDB:7MBY). **c, d** Interactions between residues in QRFPR (**c,** TMs 2-7; **d,** ICL2) and residues in the $G\alpha_q$ subunit. Hydrogen bonds are marked with red dashed lines. The elongated cytoplasmic ends of TM5 and TM6 in QRFPR relative to that in ET_BR and CCK1R are circled in **a** and **c**.

Supplementary Table S1. Cryo-EM data collection, model refinement and validation statistics for the 26RFa-QRFPR-G_q protein complex.

	26RFa-QRFPR-G_q protein-scFv16 complex<br>PDB ID: 8WZ2<br>EMD ID: EMD-37944
**Data collection and processing**	
**Magnification**	165,000
**Voltage (kV)**	300
**Electron exposure ($e^-/Å^2$)**	50
**Defocus range (μm)**	-0.8 ~ -1.8
**Pixel size (Å)**	0.73
**Symmetry imposed**	C1
**Initial particle projections (no.)**	2,397,272
**Final particle projections (no.)**	202,884
**Map resolution (Å)**	2.73
**FSC threshold**	0.143
**Map resolution range (Å)**	2.0-4.0
**Refinement**	
**Initial model used (PDB code)**	
**Model resolution (Å, FSC=0.5)**	3.20
**Model composition**	
**Non-hydrogen atoms**	9,355
**Protein residues**	1,202
**Ligand**	0
**Water**	0
***B* factors ($Å^2$, min/max/mean)**	
**Protein**	30.00/126.95/74.64
**Ligand**	-/-/-
**Water**	N/A
**R.m.s. deviations**	
**Bond lengths (Å)**	0.005
**Bond angles (°)**	0.976
**CC (mask)**	0.69
**Validation**	
**MolProbity score**	1.23
**Clashscore**	4.53
**Rotamer outliers (%)**	0.10
**Ramachandran plot**	
**Favored (%)**	98.98
**Allowed (%)**	1.02
**Disallowed (%)**	0.00

Supplementary Table S2. Cell surface expression of QRFPR and its mutants on 26RFa-induced calcium mobilization assay.

	pEC50 [a]	*Emax* [a]	Surface Expression (Relative to WT) [a]
WT	7.97 ± 0.05	100 ± 1.0	100 ± 3.6
Δ1-18	5.56 ± 0.06	53.2 ± 0.75	29.6 ± 5.4
Δ1-38	NA [b]	14.7 ± 1.5	32.7 ± 2.6
$C98^{2.57}A$	6.72 ± 0.09	48.4 ± 0.88	38.7 ± 1.5
$V101^{2.59}A$	7.97 ± 0.05	102 ± 1.7	105 ± 3.6
$T102^{2.60}A$	8.02 ± 0.07	94.0 ± 1.4	92.6 ± 4.3
$L104^{2.62}A$	8.07 ± 0.04	109 ± 0.69	81.3 ± 3.7
$Q105^{2.63}A$	7.87 ± 0.08	83.8 ± 3.1	53.1 ± 1.0
$S108^{2.65}A$	7.76 ± 0.10	51.7 ± 0.73	66.1 ± 4.9
$D109^{2.67}A$	7.93 ± 0.01	111 ± 1.3	98.3 ± 3.6
$N110^{23.49}A$	8.04 ± 0.02	98.6 ± 2.3	62.4 ± 4.6
$W111^{23.50}A$	5.34 ± 0.03	73.4 ± 1.7	56.6 ± 3.5
$I117^{3.24}A$	8.2 ± 0.19	75.1 ± 3.1	110 ± 5.7
$C118^{3.25}A$	NA	1.43 ± 0.55	17.0 ± 5.1
$V121^{3.28}A$	7.4 ± 0.09	106 ± 5.4	85.2 ± 5.8
$P122^{3.29}A$	8.33 ± 0.09	96.7 ± 1.4	61.8 ± 4.7
$Q125^{3.32}A$	5.65 ± 0.75	14.1 ± 1.8	11.3 ± 6.8
$S126^{3.33}A$	7.9 ± 0.06	92.3 ± 4.1	44.1 ± 5.6
$V129^{3.36}A$	7.22 ± 0.16	42.7 ± 0.71	20.0 ± 3.1
$M180^{4.60}A$	7.99 ± 0.12	93.3 ± 1.0	71.1 ± 7.4
$Q184^{4.64}A$	6.93 ± 0.10	71.4 ± 2.2	62.4 ± 4.1
$K189^{ECL2}A$	7.65 ± 0.08	94.9 ± 1.6	29.8 ± 2.6
$Y190^{ECL2}A$	6.25 ± 0.16	23.6 ± 0.22	22.4 ± 1.9
$F192^{ECL2}A$	7.65 ± 0.06	87.4 ± 0.93	65.5 ± 3.0
$L193^{ECL2}A$	6.66 ± 0.01	66.8 ± 0.54	41.5 ± 6.7
$I199^{ECL2}A$	7.92 ± 0.01	89.7 ± 2.3	75.4 ± 7.0
$C200^{ECL2}A$	7.74 ± 0.09	88.5 ± 1.2	132 ± 1.4
$C201^{ECL2}A$	NA	1.84 ± 1.5	26.3 ± 2.8
$L202^{45.51}A$	7.77 ± 0.03	101 ± 2.3	91.1 ± 4.6
$E203^{45.52}A$	6.12 ± 0.02	96.9 ± 0.64	88.1 ± 3.6
$W205^{ECL2}A$	NA	2.58 ± 1.2	25.1 ± 3.0
$Q211^{5.35}A$	7.64 ± 0.15	94.9 ± 1.6	72.8 ± 1.5
$Y214^{5.38}A$	NA	1.42 ± 0.70	21.7 ± 2.5
$T215^{5.39}A$	7.82 ± 0.04	85.8 ± 3.4	82.1 ± 4.7
$I218^{5.42}A$	7.68 ± 0.06	77.8 ± 0.31	53.1 ± 1.4
$L222^{5.46}A$	8.1 ± 0.02	99.0 ± 1.3	86.9 ± 2.6
$W286^{6.48}A$	NA	1.80 ± 0.38	29.7 ± 1.6
$F289^{6.51}A$	5.7 ± 0.09	54.4 ± 1.4	42.3 ± 2.4
$F289^{6.51}L$	7.73 ± 0.12	112 ± 5.1	63.7 ± 2.3
$H290^{6.52}A$	6.98 ± 0.09	60.4 ± 0.39	91.4 ± 6.6
$H293^{6.55}A$	7.8 ± 0.08	95.5 ± 1.6	65.5 ± 2.6
$E297^{6.59}A$	7.49 ± 0.05	107 ± 0.80	182 ± 4.1
$D307^{7.27}A$	7.93 ± 0.04	106 ± 0.64	171 ± 2.0
$Q318^{7.39}A$	NA	1.69 ± 0.66	27.5 ± 7.1
$F322^{7.43}A$	NA	11.1 ± 0.26	29.0 ± 2.1

[a] Data shown are means ± S.E.M. from at least three independent experiments.

[b] NA indicates that the activation level is too low to determine EC_{50} values.

Supplementary Table S3. Cell surface expression of NPFF1R and its mutants on NPVF-induced calcium mobilization assay.

	pEC_{50} [a]	*Emax* [a]	Surface Expression (Relative to WT) [a]
WT	7.45 ± 0.09	100 ± 15	100 ± 5.3
$Q123^{3.32}A$	5.22 ± 0.40	28.2 ± 4.7	40.2 ± 3.4
$E205^{45.52}A$	NA [b]	30.9 ± 4.8	51.8 ± 2.5
$D294^{6.59}A$	5.98 ± 0.45	32.0 ± 2.6	47.2 ± 2.0
$H315^{7.39}A$	NA	5.96 ± 4.1	53.7 ± 3.7

[a] Data shown are means ± S.E.M. from at least three independent experiments.

[b] NA indicates that the activation level is too low to determine EC_{50} values.

Supplementary Table S4. Cell surface expression of NPFF2R and its mutants on NPFF-induced calcium mobilization assay.

	pEC50 [a]	*Emax* [a]	Surface Expression (Relative to WT) [a]
NPFF2R-WT	7.59 ± 0.2	100 ± 8.3	100 ± 2.5
$Q125^{3.32}A$	7.18 ± 0.25	17.9 ± 3.2	86.3 ± 3.2
$E208^{45.52}A$	NA [b]	9.01 ± 1.3	95.9 ± 2.7
$D298^{6.59}A$	5.45 ± 0.15	103 ± 7.3	112 ± 2.5
$H319^{7.39}A$	6.55 ± 0.23	49.1 ± 3.4	81.8 ± 3.1

[a] Data shown are means ± S.E.M. from at least three independent experiments.

[b] NA indicates that the activation level is too low to determine EC_{50} values.

Supplementary Table S5. Cell surface expression of PrRPR and its mutants on PrRP-20-induced calcium mobilization assay.

	pEC50 [a]	*Emax* [a]	Surface Expression (Relative to WT) [a]
WT	8.96 ± 0.09	100 ± 3.9	100 ± 2.7
Q141$^{3.32}$A	8.98 ± 0.09	49.2 ± 1.2	95.6 ± 3.0
E213$^{45.52}$A	6.94 ± 0.08	27.7 ± 0.37	61.5 ± 4.1
D302$^{6.59}$A	7.95 ± 0.09	124 ± 8.9	114 ± 4.3
H321$^{7.39}$A	7.83 ± 0.32	19.7 ± 0.75	24.0 ± 0.80

[a] Data shown are means ± S.E.M. from at least three independent experiments.

Supplementary Table S6. Cell surface expression of KISS1R and its mutants on KP-10-induced calcium mobilization assay.

	pEC50 [a]	*Emax* [a]	Surface Expression (Relative to WT) [a]
WT	8.37 ± 0.10	100 ± 1.2	100 ± 5.9
Q122$^{3.32}$A	8.29 ± 0.03	12.8 ± 0.76	90.0 ± 3.8
E193$^{45.52}$A	8.00 ± 0.11	126 ± 6.2	128 ± 6.1
H309$^{7.39}$A	7.76 ± 0.04	89.6 ± 1.0	62.8 ± 2.7

[a] Data shown are means ± S.E.M. from at least three independent experiments.
